# Adapting a Two-Photon Scanning Microscope for Simultaneous Single-Photon Imaging of an Infrared Dopamine Sensor

**DOI:** 10.1523/ENEURO.0010-26.2026

**Published:** 2026-05-05

**Authors:** Matthew Tarchick, Franklin Caval-Holme, Ben Smith, Petra Mocellin, Markita Landry, Natsumi Komatsu, Marla B. Feller

**Affiliations:** ^1^Department of Neuroscience and Helen Wills Neuroscience Institute, University of California, Berkeley, Berkeley, California 94720; ^2^School of Optometry, University of California, Berkeley, Berkeley, California 94720; ^3^California Institute for Quantitative Biosciences, University of California, Berkeley, Berkeley, California 94720; ^4^Department of Chemical & Biomolecular Engineering, University of California, Berkeley, Berkeley, California 94720; ^5^Department of Bioengineering, University of Illinois Urbana-Champaign, Urbana, Illinois 6180

## Abstract

We describe a novel method for adapting a two-photon scanning microscope to enable simultaneous detection of two-photon-generated visible fluorescence and single-photon-generated near-infrared (nIR) fluorescence. In this configuration, nIR fluorescence is routed through a single-mode optical fiber before detection by a photomultiplier tube. This fiber coupling offers two advantages: first, the optical fiber functions as a pinhole aperture, allowing for improved optical sectioning of the nIR signal; second, it minimizes nIR background fluorescence. To validate the effectiveness of this design, we conducted two sets of experiments in male and female C57B/6J mice. First, we compare two fluorescence indicators of the neurotransmitter dopamine: the genetically encoded indicator GRAB_DA_ and single-walled carbon nanotube-based optical nanosensors (nIRCats). Although nIRCats exhibit lower affinity for dopamine than GRAB_DA_, this property allows for identification of high concentration release sites in the striatum. Second, we simultaneously imaged depolarization-induced calcium changes and dopamine release in the retina. Together, these results demonstrate the utility of integrating confocal nIR detection into a two-photon platform for simultaneous functional imaging across complementary spectral channels.

## Significance Statement

Dual-color, real-time imaging is a powerful technique in biomedical imaging, including neuroscience. Here, we present a widely applicable modification to a standard two-photon scanning microscope that adds a near-infrared detection capability, a wavelength range that minimizes photon scattering and autofluorescence from biological samples. Using this microscope, we demonstrate the first direct comparison of two dopamine sensors: the genetically encoded sensor GRAB_DA3m_ detected in the visible channel and carbon nanotube-based sensors detected in the near-infrared channel. We further demonstrate simultaneous imaging of calcium activity and dopamine signaling in the developing retina. While we focused on dopamine sensors in this study, this platform is broadly applicable to a wide range of fluorophores and can be implemented on existing two-photon microscopes.

## Introduction

The neuromodulator dopamine plays a critical role in multiple brain functions (Speranza et al., 2021). Understanding the mechanisms by which dopamine impacts brain activity requires measuring spatial and temporal dynamics of extracellular dopamine concentrations (Sippy and Tritsch, 2023). For example, diverse firing patterns in the ventral midbrain modulate distinct brain functions: tonic firing in substantia nigra dopaminergic neurons produces sustained elevation of dopamine in the dorsal striatum that promotes a permissive environment for movement (da Silva et al., 2018), while burst firing in the ventral tegmental area generates transient elevations of dopamine associated with reward prediction errors (Bayer and Glimcher, 2005). In the retina, dopamine similarly acts as a critical neuromodulator, regulating short-term light adaptation, modulating receptive field properties, and contributing to circadian changes in retinal function (Witkovsky, 2004; Jackson et al., 2012; Roy and Field, 2019).

To characterize the dynamics of dopamine release, the field has turned to optical sensors. One popular class of these sensors includes genetically encoded sensors in which green fluorescent proteins are integrated with dopamine receptors (dLight and GPCR activation-based dopamine sensors GRAB_DA_; Patriarchi et al., 2018; Sun et al., 2018). These sensors have proven to be powerful detectors of dopamine release but require viral transduction of the sensors and therefore are limited to use in genetically tractable organisms.

Another tool for detecting dopamine optically is synthetic near-infrared (nIR) catecholamine nanosensors (nIRCat) (Beyene et al., 2019; Del Bonis-O'Donnell et al., 2021). nIRCat nanosensors are synthesized from single-walled carbon nanotubes, which fluoresce in the near-infrared, a window that prevents scattering in tissue. They are functionalized with (GT)_6_ DNA sequences to have specificity for dopamine and norepinephrine over other neurotransmitters. nIRCat sensors have a lower affinity for dopamine than the genetically encoded sensors (Beyene et al., 2019), but they allow for the detection of hotspots of dopamine release, such as in slices of the dorsal striatum. In addition, nIRCats have been incorporated into films, allowing for the characterization of dopamine release along isolated dopaminergic axons (Bulumulla et al., 2022; Elizarova et al., 2022).

Furthermore, nIRCat's near-infrared fluorescence is spectrally well separated from visible fluorophores, providing an opportunity to study dopamine release simultaneously with calcium dynamics or other neurotransmitter activities. Dual imaging of nIRCat and visible wavelength fluorophores has been demonstrated (Bulumulla et al., 2022; Elizarova et al., 2022); however, visible wavelength fluorophores were used to label neurons in those studies, rather than to monitor real-time activity.

Here we introduce a dual-modal scanning microscope that integrates two-photon excitation of a visible fluorophore with single-photon excitation of nIR nanosensors. Optical channels are spectrally resolved and independently coupled to maximize signal fidelity. We validated our design by measuring real-time dopamine dynamics in the striatum with simultaneous imaging of nIRCats and GRAB_DA3m_. We further demonstrate real-time imaging of depolarization-induced calcium transients and simultaneous dopamine release in the retina. While we focus on monitoring sensors of Ca^2+^ and neurotransmitter release as a proof of concept in this study, this framework generalizes to any visible fluorophores and supports flexible expansion to other analytes or modalities.

## Materials and Methods

### Experimental model

All experiments were performed in male and female C57B/6J mice (Jackson Laboratory). The animals were maintained on a 12 h light cycle (lights on at 07:00). All animal procedures were performed in accordance with the University of California Berkeley Institutional Animal Care committee's regulations and Use Committees and conformed to the National Institutes of Health's Guide for the care and use of laboratory animals, the Public Health Service Policy, and the Society for Neuroscience Policy on the Use of Animals in Neuroscience Research.

### Stereotactic viral injection

The injections were performed in 6–8 week-old mice under general ketamine–dexmedetomidine anesthesia using a stereotaxic instrument (Kopf Instruments, Model 1900) as previously described (Lammel et al., 2008). For the GRAB_DA_ experiment, 400 nl of AAV2/9-hSyn-GRAB_DA3m-WPRE-hGH polyA (BrainVTA, PT-4720) was injected bilaterally in the dorsal striatum. The coordinates used to target the dorsal striatum were AP: +1; ML: ±1.5; DV: −3.0 relative to bregma and based on the Allen Mouse Brain Atlas. After all injections, the needle was left in place for at least 5 min before withdrawal. The animals were kept on a heating pad until recovered from anesthesia. Experiments were performed 3–4 weeks after stereotactic injection.

### Acute brain slice preparation

Three to four weeks after viral injection, mice (10–12 weeks old) were deeply anaesthetized with pentobarbital (200 mg/kg, i.p.; Vortech). After intracardial perfusion with ice-cold artificial cerebrospinal fluid [aCSF; containing the following (in mM): 119 NaCl, 26.2 NaHCO_3_, 2.5 KCl, 1 NaH_2_PO_4_, 3.5 MgCl_2_, 10 glucose, and 0 CaCl_2_], the brain was rapidly extracted and sliced with a Leica VT1200 S Vibratome. Before use, slices were incubated at 37°C for 60 min in oxygen-saturated aCSF (in mM: 119 NaCl, 26.2 NaHCO_3_, 2.5 KCl, 1 NaH_2_PO_4_, 1.3 MgCl_2_, 10 glucose, and 2 CaCl_2_) and then transferred to room temperature for 30 min before nIRCat labeling and imaging experiments.

### Retina preparation

Postnatal day (P) 4–8 mice were deeply anesthetized with isoflurane inhalation and killed by decapitation. Eyes were immediately enucleated, and retinas were dissected in oxygenated (95% O_2_, 5% CO_2_) aCSF (in mM: 119 NaCl, 2.5 KCl, 1.3 MgCl_2_, 1 K_2_HPO_4_, 26.2 NaHCO_3_, 11 d-glucose, and 2.5 CaCl_2_) at room temperature under white light. In some experiments, 0.1 mM sulforhodamine 101 (SR101, Invitrogen) was added for visualization of vasculature. Each isolated retina was cut into two pieces. Each piece of retina was mounted over a 1–2 mm^2^ hole in nitrocellulose filter paper (Millipore) with the photoreceptor layer side down, dark-adapted for 1 h, and transferred to the recording chamber of a two-photon microscope for imaging. The whole-mount retinas were continuously perfused (3 ml/min) with oxygenated aCSF warmed to 32–34°C by a regulated inline heater (TC-344B, Warner Instruments) for the duration of the experiment. Additional retina pieces were kept in the dark at room temperature in aCSF bubbled with 95% O_2_, 5% CO_2_ until use (maximum 8 h).

### nIRCat synthesis

nIRCats were prepared following a previously developed protocol (Beyene et al., 2019). Briefly, HiPCo SWCNT slurry (NanoIntegris) and ssDNA (Integrated DNA Technologies) were mixed with a final NaCl concentration of 10 mM. The mass ratio of DNA to SWCNT was 2:1. The DNA sequence was GTGTGTGTGTGT. The mixture was probe-tip sonicated (Cole-Parmer Ultrasonic Processor, 3 mm tip) in ice-cold water for 30 min at 50% amplitude. The resulting suspensions were centrifuged at 21,000 × *g* for 4 h at 4°C and the supernatant was collected. Nanosensors were diluted to 200 mg/L in 10 mM NaCl and stored at 4°C.

### nIRCat labeling (brain slices)

aCSF (in mM: 119 NaCl, 26.2 NaHCO_3_, 2.5 KCl, 1 NaH_2_PO_4_, 1.3 MgCl_2_, 10 glucose, 2 CaCl_2_, all purchased from Sigma-Aldrich) was prepared and bubbled with carbogen gas (oxygen/carbon dioxide 95% O_2_, 5% CO_2_, Praxair). To label the slice with nIRCat, brain slices containing striatum were transferred to an incubation chamber filled with carbogen-bubbled aCSF at room temperature. nIRCats were applied to the surface of brain slices with a pipette to a final concentration of 2 mg/L and incubated for 15 min. Slices were rinsed for 5 s with bubbled aCSF through 3 wells of a 24-well plate to remove unlocalized nanosensors and rested for at least 15 min before imaging.

### nIRCat and Cal 520 labeling (retina)

Retinas were transferred to the recording chamber of a two-photon microscope. nIRCat sensors (10 mg/ml in aCSF) were combined 1:1 with Cal 520 AM (AAT Bioquest) and bulk-loaded into the retina using a multicell bolus loading technique (Stosiek et al., 2003; Blankenship et al., 2009). Bolus loading was targeted to the retina's inner plexiform layer so as to deposit the sensor near processes of dopaminergic amacrine cells. Retinas were allowed to equilibrate for 1 h before imaging.

### Imaging

Samples were imaged on a modified two-photon microscope, where a near-infrared detection arm was added to the descanned laser path, allowing for simultaneous single-photon near-infrared confocal imaging (design files with parts list are provided here: https://github.com/Llamero/Two-Photon_nIR_descanned_confocal_arm). Carbogen-bubbled aCSF was perfused through the microscope chamber. Slices labeled with nIRCats were placed in the chamber with a tissue harp, and a glass pipet or a bipolar stimulation electrode (MicroProbes for Life Science Stereotrodes Platinum/Iridium Standard Tip) was positioned to the targeted field of view, which was adjusted with a 4× objective followed by a 60× objective (Olympus 1.00 NA LUMPlanFLN). The microscope was controlled by ScanImage Software. Fluorescent images were acquired at a frame rate of 1.47 frames/s in a 130 µm^2^ (256 × 256 pixel) field of view. An ultrafast pulsed laser (tuned to 920−950 nm) provided fluorescence excitation.

To measure depolarization-evoked dopamine release, 1 s of 100 mM potassium solution or 1 ms of 0.1–0.3 mA stimulation was applied, and this stimulation was repeated at least three times with the same field of view, with 5 min between each stimulation. For the pharmacological measurements, 10 µM of quinpirole (Fisher Scientific, 10-611-0) was applied to the chamber through aCSF perfusion, and slices were incubated for 15 min before imaging. Images were acquired in the same field of view as the one before quinpirole application.

### Image analysis—striatal GRAB_DA_ and nIRCat imaging

Imaging movie files were processed using a Python script (https://github.com/NicholasOuassil/NanoImgPro). Fluorescent modulation Δ*F*/*F*_0_ was calculated as Δ*F*/*F* = (*F* − *F*_0_) / *F*_0_, where *F*_0_ is the average intensity before the stimulation and *F* is the dynamic fluorescence intensity from the entire field of view. Next, an 8 × 8 pixel (corresponding to 6 µm by 6 µm) grid mask was applied to the image stack to minimize bias and improve stack processing time. For each grid square, Δ*F*/*F*_0_ was calculated, and they were identified as a region of interest (ROI) if the Δ*F*/*F*_0_ after stimulation was 2 standard deviations above the baseline activity. All the parameters reported in this study are averaged over at least three stimulations.

To compare the time course of the fluorescence responses for nIRCats and GRAB_DA3m_, the Δ*F*/*F*_0_ data was fitted to the following:

$$\alpha \times \left(1-\exp\left(-\frac{x}{\tau_{\mathrm{ON}}}\right)\right)\times \left(\exp\left(-\frac{x}{\tau_{\mathrm{OFF}}}\right)\right)+\beta ,$$

where α is a scale factor, β is a constant, *τ*_ON_ is the rise time constant, and *τ*_OFF_ is the decay time constant. α, β, *τ*_ON_, and *τ*_OFF_ were fitting parameters.

### Image analysis—retinal Ca^2+^ and nIRCat imaging

For each field of view, a single ROI was defined and fluorescence data extracted from both spectral channels in FIJI. ROIs encompassed the entire field of view or the region labeled with nIRCat sensor. Wave-triggered and K^+^ puff-triggered average responses were computed using a Python script (https://github.com/FellerLabCodeShare/nIR-catecholamine-sensor). Fluorescence signals were low-pass filtered at 0.68 Hz using a third-order Butterworth filter. Fluorescent modulation Δ*F*/*F*_0_ was calculated as Δ*F*/*F* = (*F* − *F*_0_) / *F*_0_, where *F*_0_ is the median intensity during the imaging trial. In some cases, nIRCat fluorescence exhibited an exponential decay during the first 30–60 s of imaging. These decays were removed by fitting and then subtracting a double exponential. Spontaneous retinal waves were identified by *Z*-scoring the Cal 520 signal and finding peaks that exceeded the mean by >3 standard deviations and were separated by at least 10 s. Wave and puff-triggered averages were computed within a 60 s window centered on the frame prior to the wave peak or K^+^ puff. Responses to puffs and waves were measured from the triggered averages by computing the difference between the peak values (a mean within a 3 s window centered on the peak) in 10 s windows before and after the event trigger.

### Code accessibility

Python-based image processing and analysis applications are available at https://github.com/NicholasOuassil/NanoImgPro and https://github.com/FellerLabCodeShare/nIR-catecholamine-sensor. The code is publicly accessible and does not require special permissions; no accession number is applicable. Analyses were performed using Python (version 3.12.12) on macOS-based systems and in cloud-based environments using Google Colaboratory. The code was executed on standard workstation hardware, and required packages and installation instructions are provided in the repository.

## Results

### Integrating an infrared detection pathway into a scanning two-photon microscope

nIRCat sensors fluoresce in the near-infrared with a broad range of excitation bands (Cambré et al., 2025). Thus far, imaging of these sensors in brain tissue has been based on epifluorescent imaging, using InGaAs cameras that are sensitive to long wavelengths (Yang et al., 2021). Here we present a strategy for integrating an infrared detection pathway into a two-photon (2P) laser scanning microscope (tuned 920–950 nm), which will allow for multiplexed imaging of these sensors with visible wavelength sensors.

**Figure 1.**
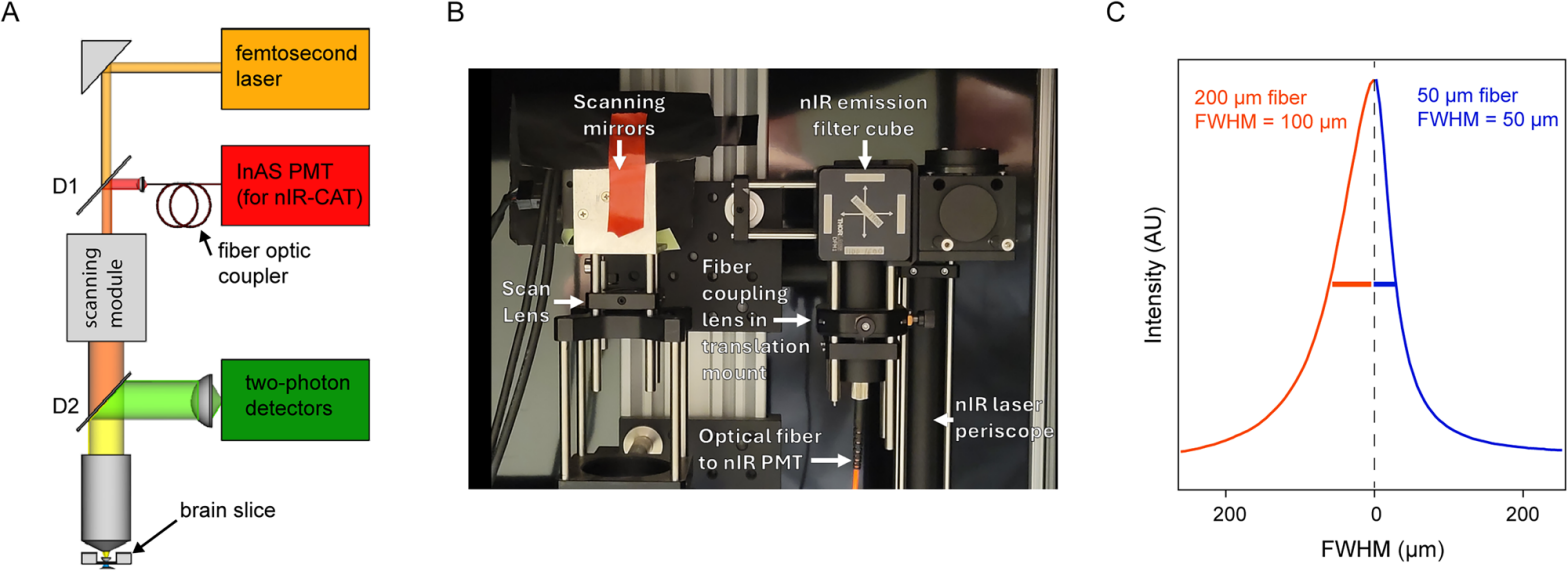
Integrating an infrared detection pathway for imaging of nIRCat fluorescence. ***A***, Microscope schematic. A one-photon infrared (IR) detection arm (red) was added to a scanning two-photon microscope. Dichroics: D1: custom dichroic is 680–1,040 short pass, reflecting >1,100 nm. D2: 680 nm long-pass. There is a 1,300 nm low-pass before the InAs PMT. ***B***, A photo of the near-infrared descanned detection arm for the two-photon microscope. ***C***, Limited *z*-section provided by the aperture of the fiber optic coupler. Fluorescence profiles of IR fluorescence from lead sulfide quantum dots for a thicker fiber (red, 200 µm) and a thinner fiber (blue, 50 µm). FWHM was 100 µm for the thick fiber and 50 µm for the thin fiber.

We integrated an indium arsenide (InAs) photomultiplier tube (PMT, Hamamatsu H12397A-75), well established for low-noise infrared detection, into a scanning 2P microscope. The InAs nIR PMT was integrated in the descanned path of the microscope, allowing for the simultaneous imaging of the 2P excitation of visible wavelength sensors and the single-photon confocal imaging of the nIRCat sensors (Fig. 1*A*,*B*). Specifically, a 1,100 nm short-pass dichroic mirror was inserted into the two-photon excitation path immediately upstream of the scanning mirrors. This dichroic transmits the two-photon excitation beam while reflecting the near-infrared (nIRCat) emission. The reflected nIR fluorescence then passes through a 1,300 nm long-pass filter to reject residual reflected excitation light before being focused into a single-mode optical fiber. The fiber acts as a confocal pinhole, rejecting out-of-focus fluorescence from above and below the focal plane and guiding the nIR signal to the InAs photomultiplier tube for detection.

To assess the *z*-sectioning obtained by the optical fiber, we imaged a thin film of lead sulfide quantum dots adhered to a glass slide using both a 50-µm-thick optical fiber and a 200-µm-thick optical fiber (Fig. 1*C*). We estimated the effective axial resolution as measured at the FWHM of the *z*-intensity profile of the resulting image stack. For thick fibers, this corresponds to 100 µm while for the 50 µm fibers it was 50 µm. This limited confocality allows for integration of IR signal over the full range of nIRCat sensors in the tissue.

### Dual real-time imaging of nIRCat and GRAB_DA_ in dorsal striatum

**Figure 2.**
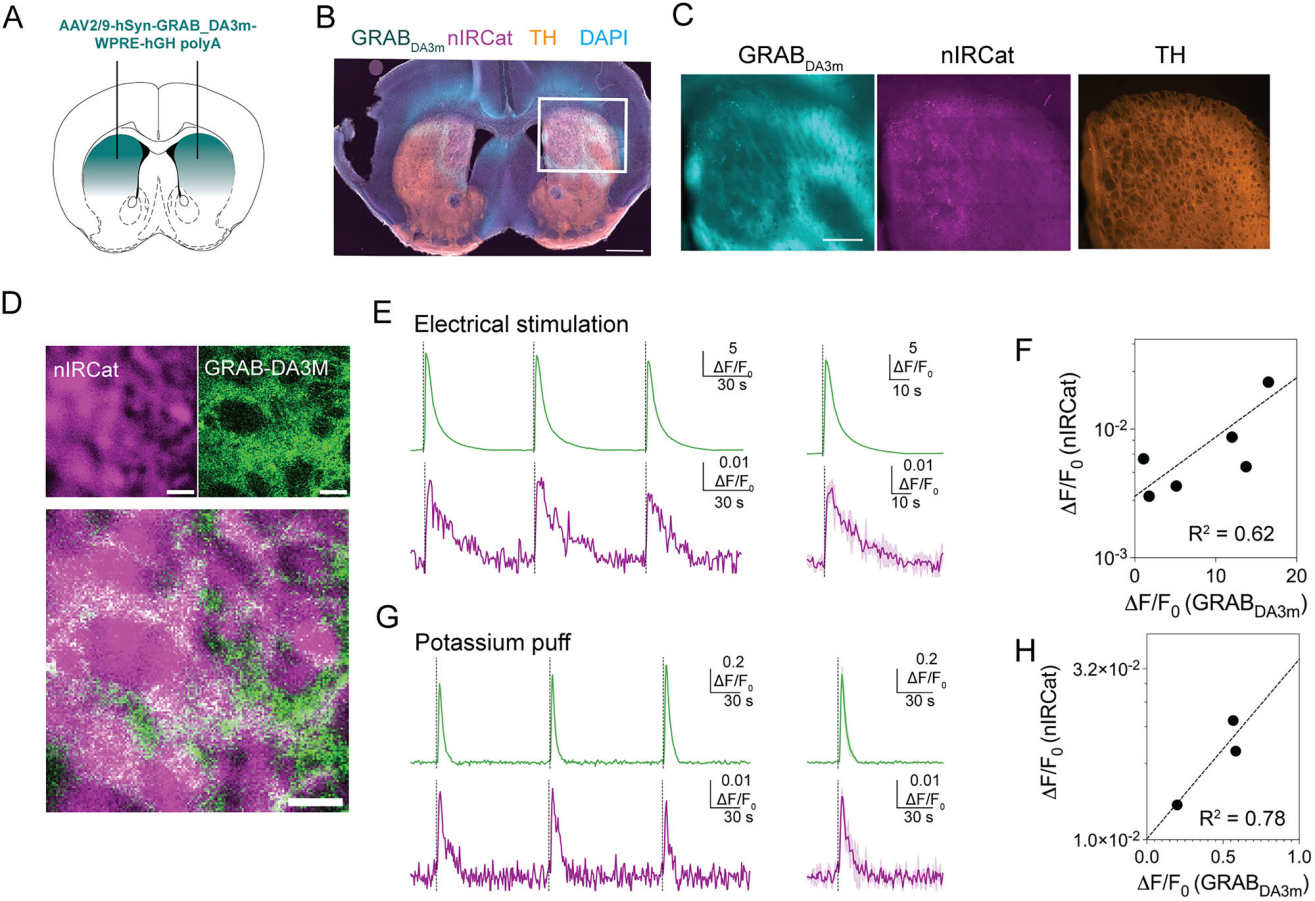
Direct comparison of response amplitudes of nIRCat and GRAB_DA3m_ to evoked dopamine release. ***A***, Scheme of the GRAB_DA3m_ injection sites in the dorsal striatum. ***B***, Representative striatal slice showing GRAB_DA3m_ viral expression in cyan, putative nIRCat signal in magenta (far red wavelength), and tyrosine hydroxylase (TH) antibody staining in orange. Scale bar, 1 mm. ***C***, Same as ***B*** at a higher magnification. Scale bar, 500 µm. ***D***, Fluorescence images from the nIR channel (nIRCat; left), from the visible channel (GRAB_DA3m_; right), and their overlay images (bottom). Scale bar denotes 20 µm. ***E***, Representative fractional change (Δ*F*/*F*_0_) in fluorescence of GRAB_DA3m_ (top, green) and nIRCat (bottom, purple), integrated from a 74 µm × 74 µm field of view, in response to depolarization via 300 µA electrical stimulation. Individual response (left) and the averaged trace from three repeated stimuli (solid) with SD (shadow) (right). ***F***, Comparison of the integrated Δ*F*/*F*_0_ between nIRCat and GRAB_DA3m_. The integrated Δ*F*/*F*_0_ was obtained from a 74 µm × 74 µm field of view. Each point is the average of three stimuli. *n* = 6 brain slices from 1 mouse. Data were fitted with linear regression (dashed line; *R*^2^ = 0.62). ***G***, Example of Δ*F*/*F*_0_ of GRAB_DA3m_ (top, green) and nIRCat (bottom, purple), integrated from a 74 µm × 74 µm field of view, in response to depolarization via potassium application (100 mM, 1 s). Individual response (left) and the averaged trace from three repeated stimuli (solid) with SD (shadow) (right). ***H***, Comparison of the integrated Δ*F*/*F*_0_ between nIRCat and GRAB_DA3m_. The integrated Δ*F*/*F*_0_ was obtained from a 74 µm × 74 µm field of view. Each point is the average of three stimuli. *n* = 3 brain slices from 1 mouse. Data were fitted with linear regression (dashed line; *R*^2^ = 0.78).

To demonstrate the dual-imaging capability of our microscope, we simultaneously imaged evoked dopamine release in acute brain slices using both nIRCats and GRAB_DA_. We prepared coronal slices from mice expressing GRAB_DA3m_ in the dorsal striatum (Fig. 2*A–C*), a region chosen for its dense dopaminergic innervation and lack of norepinephrine inputs (Berridge and Waterhouse, 2003) as nIRCats are also sensitive to norepinephrine (Beyene et al., 2019; Del Bonis-O'Donnell et al., 2021). We then incubated slices with nIRCats for 15 min before imaging. Using both visible and nIR channels, we observed robust fluorescent signals in both sensors (Fig. 2*D*). We note that the spatial distribution of GRAB_DA3m_ and nIRCat did not completely overlap (Fig. 2*D*), likely because GRAB_DA3m_ is expressed on cell membranes (Sun et al., 2018) whereas nIRCats reside in the brain extracellular space.

We next electrically evoked dopamine release to assess the microscope's dual, real-time imaging performance. Depolarization with bipolar stimulating electrodes evoked increases in fluorescence (Δ*F*/*F*_0_) in sensors; both GRAB_DA3m_ and nIRCat responded reliably to repeated stimulation (Fig. 2*E*), confirming their reversibility.

We directly compared the sensor performance of nIRCats and GRAB_DA3m_: Δ*F*/*F*_0_ of nIRCats monotonically increased with Δ*F*/*F*_0_ of GRAB_DA3m_ (Fig. 2*F*). The Δ*F*/*F*_0_ of nIRCats was approximately three orders of magnitude smaller than that of GRAB_DA3m_, consistent with the sensors’ different affinities [estimated to be tens of µM for nIRCats (Beyene et al., 2019) and tens of nM for GRAB_DA3m_ (Zhuo et al., 2024)]. In addition to electrical stimulation, both sensors showed robust responses to short applications of potassium (K^+^; Fig. 2*G*,*H*). The nIRCat Δ*F*/*F*_0_ values exhibited slightly higher variability than GRAB_DA3m_ across brain slices, likely due to changes in loading and the lower signal-to-noise. Note that the Δ*F*/*F*_0_ magnitude of nIRCats imaged here was one order smaller than prior reports (Beyene et al., 2019; Del Bonis-O'Donnell et al., 2021), likely because of differences in excitation and detection settings and differences in detector quantum efficiency above 1,100 nm.

**Figure 3.**
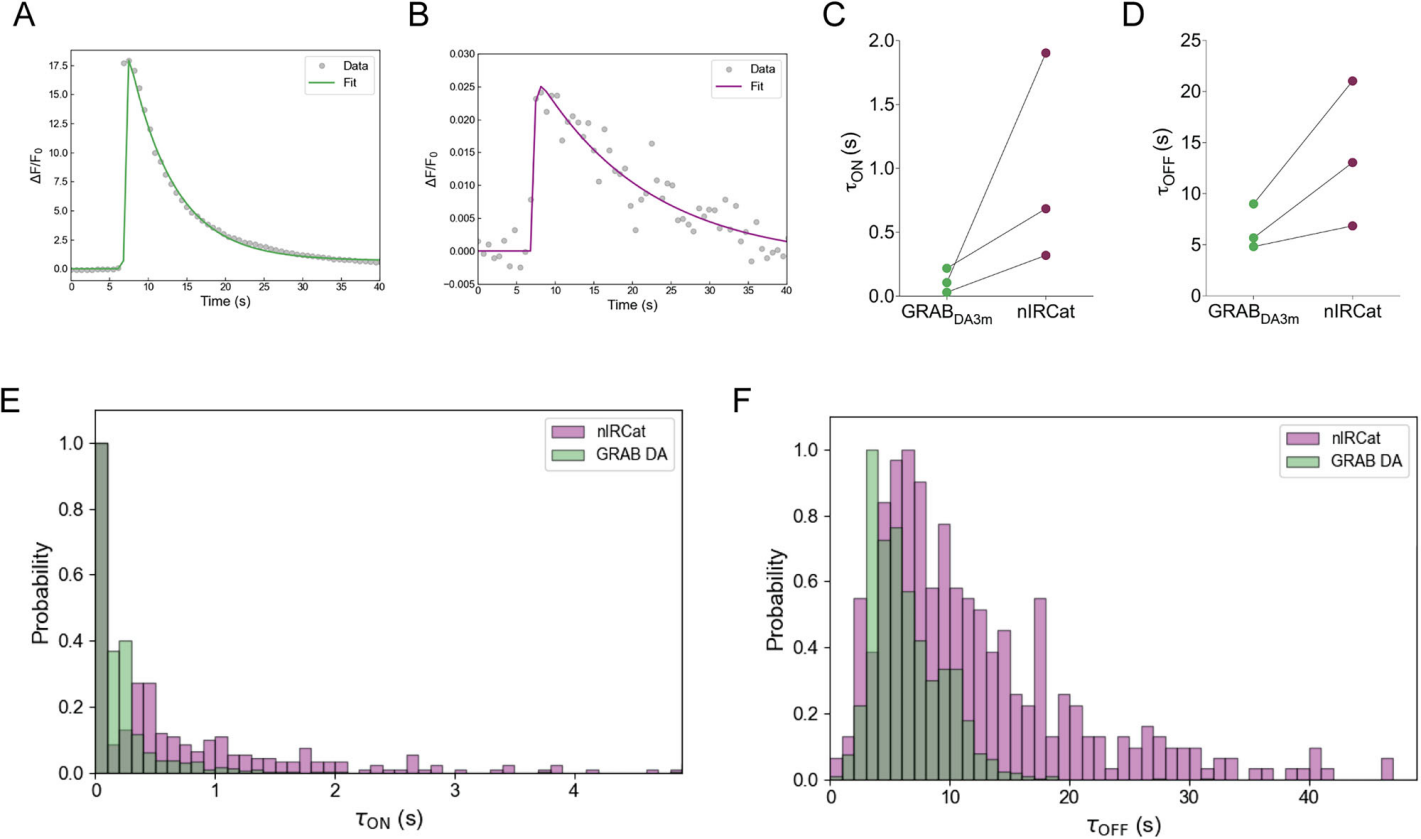
Comparison of response kinetics of nIRCat and GRAB_DA3m_ to evoked dopamine release. ***A***, Example average responses of GRAB_DA3m_ to electrically evoked dopamine release in striatal slices and their fits used to obtain *τ*_ON_ and *τ*_OFF_. ***B***, Same as ***A*** for nIRCat. ***C***, Summary data comparing *τ*_ON_ for GRAB_DA3m_ and nIRCat for responses averaged over the entire field of view (74 µm × 74 µm). Each point is the average of three stimuli. *n* = 3 brain slices from 1 mouse. ***D***, Same as ***C*** for *τ*_OFF_. ***E***, Distributions of *τ*_ON_ for GRAB_DA3m_ (green) and for nIRCat (pink) for local ROIs (6 µm × 6 µm). ***F***, Same as ***E*** for *τ*_OFF_.

We further analyzed the time constants of both sensors (Fig. 3). By analyzing integrated Δ*F*/*F*_0_, averaged over the entire field of view (74 µm × 74 µm; Fig. 3*A*,*B*), nIRCat signals exhibit a consistently longer rise time and decay time (Fig. 3*C*,*D*). To examine the variability of the response kinetics across the sample, we computed the time constant of the responses within a 6 × 6 µm grid mask. While the modes of both sensors overlap for *τ*_ON_ and are comparable for *τ*_OFF_, nIRCat responses displayed greater heterogeneity (Fig. 3*E*,*F*), consistent with the previous study (Beyene et al., 2019). This difference in kinetics likely reflects their volumetric sampling of dopamine throughout the extracellular space, combined with the more diffuse optical sectioning in our one-photon imaging. To our knowledge, this study provides the first direct comparison between GRAB_DA3m_ and nIRCat performance under identical experimental conditions.

### nIRCat's lower affinity enables spatial mapping of high dopamine release sites in dorsal striatum

The dorsal striatum is densely innervated by dopaminergic neurons, resulting in a high extracellular dopamine concentration upon depolarization. Although the high affinity of GRAB_DA3m_ is ideal for many applications, including in vivo imaging, it presents a limitation in dopamine-rich regions where the sensor can become saturated, compromising its suitability for spatially resolved analyses. Note, GRAB_DA_ sensors with differing affinities have been introduced to address this issue (Zhuo et al., 2024), but their *K_d_* is still in the nM range. In contrast, the lower affinity of nIRCats allows for identification of localized areas of high dopamine release, including subcellular somatodendritic release as reported earlier (Bulumulla et al., 2022).

**Figure 4.**
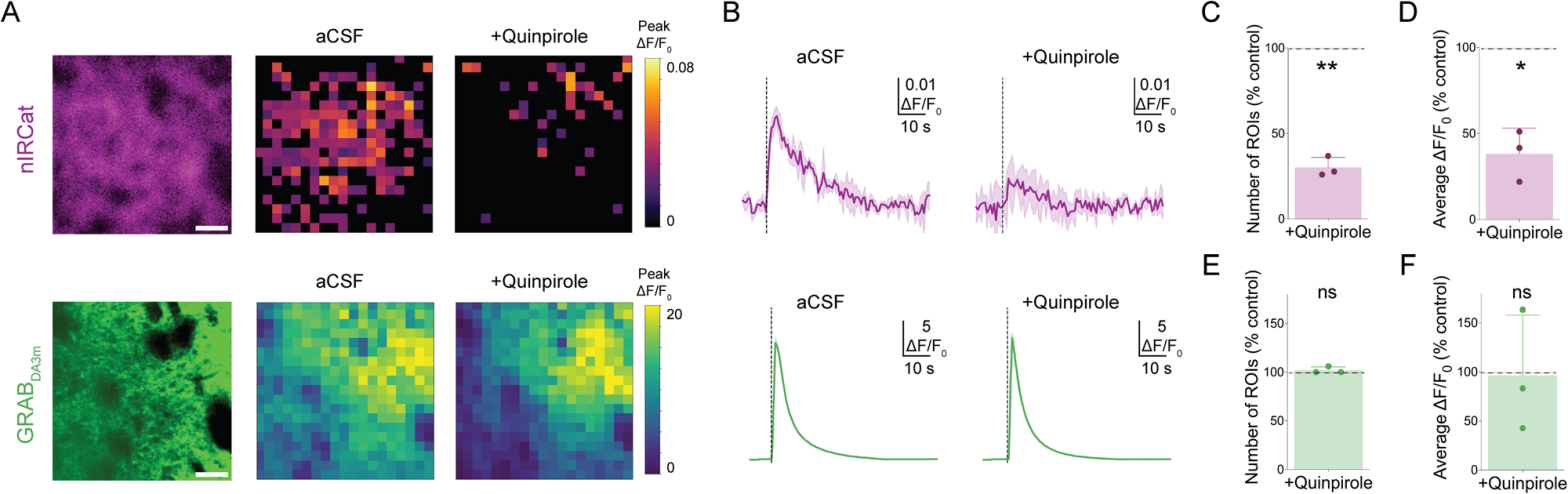
Use of nIRCat to identify localized high dopamine release sites. ***A***, Left, Fluorescent signal from nIRCat (top) and GRAB_DA3m_ (bottom). Scale bar, 20 µm. Middle, Heat map of Δ*F*/*F* responses to 300 µA electrical stimulation in 6 µm × 6 µm ROIs in aCSF. Right, Heat map after 10 µM quinpirole application. ***B***, Example of Δ*F*/*F*_0_ from the entire field of view. Average of *n* = 3 stimuli (solid) with SD (shadow). ***C***, The percent of ROIs significantly above threshold after application of 10 µM quinpirole in the nIRCat channel. Each data point is the average of three stimuli. *n* = 3 brain slices from 1 mouse. *p* = 0.0023 by paired *t* test (*t*_(2)_ = 20.68). ***D***, Same as ***C*** for average Δ*F*/*F*_0_ from the entire field of view (74 µm × 74 µm) in the nIRCat channel. *p* = 0.0188 by paired *t* test (*t*_(2)_ = 7.194). ***E***, Same as ***C*** for GRAB_DA3m_. *p* = 0.4226 by paired *t* test. ***F***, Same as ***D*** for GRAB_DA3m_ *p* = 0.9294 by paired *t* test. *p ≤ 0.05, **p ≤ 0.01, ns = not significant.

To identify putative dopamine release sites, we applied a 6 × 6 µm grid mask to the images from both GRAB_DA3m_ and nIRCat channels and calculated Δ*F*/*F*_0_ for each grid square (Fig. 4*A*). Regions of interests (ROIs) were defined as grid squares where peak Δ*F*/*F*_0_ exceeded two standard deviations above baseline. Using this approach, nIRCat imaging identified ∼150 ROIs (out of 361 grid squares) corresponding to “high dopamine spots” (Fig. 4*A*). In contrast, all 361 ROIs were active in the GRAB_DA3m_ images, consistent with the sensor's high affinity and therefore saturation. Next, to assess the sensitivity of these ROIs to changes in dopamine concentration, we applied quinpirole, a D2 dopamine receptor agonist known to decrease dopamine release. Note GRAB_DA3m_ is insensitive to quinpirole because it is based on a D1 receptor scaffold and can thus reliably be used to measure dopamine modulation in conjunction with quinpirole (Zhuo et al., 2024). Quinpirole significantly reduced the nIRCat responses by ∼70% in the number of identified ROIs and ∼60% in the integrated Δ*F*/*F*_0_ (Fig. 4*B–D*). GRAB_DA3m_ exhibited no significant change in the number of ROIs or in the integrated Δ*F*/*F*_0_ (Fig. 4*E*,*F*), consistent with sensor saturation. These results highlight that the lower-affinity nIRCat sensor can spatially resolve localized dopamine hotspots, providing a useful tool for mapping high dopamine release microdomains and assessing how these patterns change with genetic manipulation (Black et al., 2025) or experimental condition (Mun et al., 2026).

### Simultaneous two-photon calcium imaging and nIR dopamine imaging in the developing retina

To demonstrate the applicability of the microscope to other visible fluorophores and other neural circuits, we performed simultaneous two-photon calcium imaging with Cal520 and dopamine imaging with nIRCats in the retina. In the retina, the sole source of dopamine is a sparse population of amacrine cells which play essential roles in both retinal development (Reis et al., 2007; Liang et al., 2023) and adult light adaptation (Jackson et al., 2012; Roy and Field, 2019; Goel and Mangel, 2021). Although we previously demonstrated that spontaneous retinal waves evoke dopamine release (Arroyo and Feller, 2016), those experiments were limited by the use of one-photon, FRET-based CNiFER sensors (Müller et al., 2014) with relatively slow kinetics. As a result, dopamine could only be detected as a bulk signal with limited temporal resolution and no ability to resolve the spatial structure of dopamine release across the retinal tissue. Dopamine release has not been imaged directly from adult retina.

**Figure 5.**
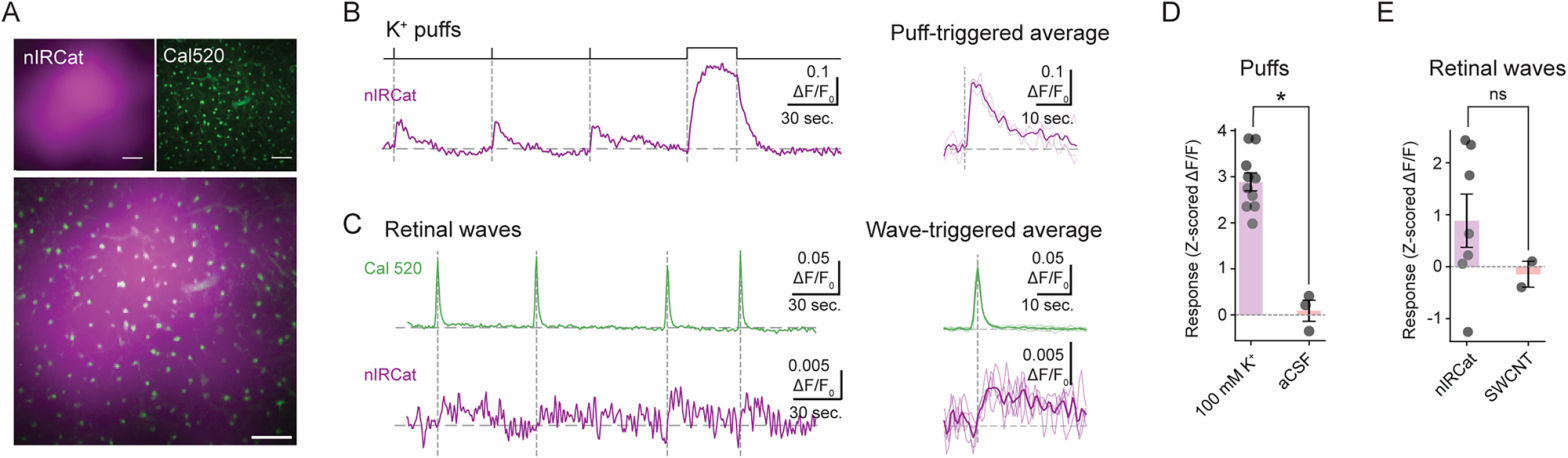
nIRCat response to dopamine release in retina. ***A***, Pseudocolor images of Cal 520 (green) and nIRCat (purple) in the retina's inner plexiform layer. Scale bars are 20 µm. ***B***, Left, Fractional change in fluorescence of nIRCat in response to dopamine release evoked by applications of K^+^ (100 mM). Pressure monitor above the traces indicates timing of puffs: three short (1 s) and one long (30 s) to probe the saturated response. Right, Puff-triggered average (thick line) and individual trials (thin lines) for the 1 s puffs. ***C***, Left, Simultaneous imaging of Cal 520 (green) and nIRCat (purple). Vertical dashed lines mark the peaks of Ca^2+^ transients associated with spontaneous retinal waves. Right, Wave-triggered averages (thick lines) and signals from individual wave events (thin lines). ***D***, Responses to puffs of 100 mM K^+^ or aCSF (a negative control). Each point is an experimental replicate (a retina piece). *n* = 7 retina pieces from 4 mice for K^+^ and *n* = 3 retina pieces from 3 mice for aCSF. Error bars represent the standard error of the mean. *p* = 0.01190 by Mann–Whitney *U* test (*t* = 18.0). ***E***, Responses to retinal waves measured after loading with dopamine-sensitive near-infrared catecholamine sensor (nIRCat) or single-walled carbon nanotubes (SWCNT; a negative control). Each point is an experimental replicate (a retina piece). *n* = 7 retina pieces from 5 mice for nIRCat and *n* = 2 retina pieces from 1 mouse for SWCNT. Error bars represent the standard error of the mean. *p* = 0.1667 by Mann–Whitney *U* test (*t* = 11.0). *p ≤ 0.05, ns = not significant.

Using our dual-imaging system—which combines near-infrared detection of the dopamine nanosensor nIRCat with two-photon calcium imaging of Cal520—we directly visualized dopamine dynamics in P4–P8 retinas (Fig. 5). Brief K^+^ puffs (100 mM, 1 s) evoked robust fractional fluorescence changes in both indicators (Fig. 5*B*), while there was no detectable change with ACSF application (Fig. 5*D*).

To assess dopamine release during retinal waves, we repeated these experiments under conditions of spontaneous activity only. Aligning nIRCat signals to spontaneous wave peaks revealed a strong temporal coupling between wave activity and dopamine release (Fig. 5*C*). However, although nIRCat responded robustly to strong depolarization evoked by K^+^ puffs, its lower dopamine affinity resulted in poor signal-to-noise for wave-evoked transients (Fig. 5*E*). Hence, the lower affinity of the nIRCat limits its ability to detect the lower dopamine concentrations associated with spontaneous retinal wave.

## Discussion

We presented a straightforward approach to introduce a nIR detection capacity to an existing two-photon microscope. This was achieved by introducing a single-mode optical fiber prior to the nIR detector without requiring an additional excitation laser. The fiber-coupled design also provided confocal capability and minimized background nIR signals. This integrated approach allows real-time comparison of dopamine dynamics measured with complementary sensors, as well as simultaneous monitoring of neuronal depolarization and neuromodulator release in intact tissue.

Introducing an nIR channel enabled simultaneous, real-time imaging of visible and nIR fluorescence. Dual-channel imaging is a powerful strategy widely used within the visible spectrum—such as concurrent recording of green and red fluorescence—to reveal complex interactions across cellular and circuit processes (Zhuo et al., 2024). In this study, we extend this capability into the near-infrared range by simultaneously monitoring nIRCat fluorescence alongside dynamically reporting genetically encoded dopamine or calcium indicators in the visible channel. While previous studies have coimaged nIRCats with neurons labeled with visible fluorophores in culture (Bulumulla et al., 2022; Elizarova et al., 2022), our approach introduces two advances: (1) functional imaging in the visible channel rather than static structural labeling and (2) confocal capability in the nIR detection by implementing fiber-based coupling, which reduces background and improves optical sectioning for imaging in tissue. Although we focused on dopamine and calcium imaging, this platform is expandable to include additional sensors. For example, synthetic organic fluorophores with emission near 1,000 nm have been developed for structural imaging (Antaris et al., 2017). Furthermore, near-infrared calcium (Qian et al., 2019) and voltage indicators (Abdelfattah et al., 2023; Han et al., 2023) have been reported, although these probes emit at shorter wavelengths (∼700 nm) and would require adjustments to the filter sets used in the current optical setup.

Multiplexed imaging of sensors with different affinities enables localization of dopamine release sites using a low-affinity sensor, while simultaneously measuring the spatial spread of physiologically relevant dopamine concentrations with high-affinity D1- or D2-based sensors. In addition, GRAB_DA_ is membrane-bound and therefore reports dopamine diffusion near the cell surface, whereas nIRCat is distributed throughout the extracellular volume, allowing measurement of the kinetics of bulk dopamine release. Together, these complementary sensors provide a unified framework for resolving both the spatial and temporal dynamics of dopamine signaling across cellular and tissue scales.

Our demonstration of simultaneous dopamine and calcium imaging in the retina showcased another potential niche for nIRCat applications, where the expression of genetically encoded sensors like GRAB_DA_ is technically challenging. Such contexts include tissues or organs where sensor expression is less characterized (e.g., retina, intestine), early developmental stages before robust expression can occur, or nontraditional model organisms lacking fully annotated genomes (e.g., meadow voles; (Mun et al., 2026)).

Live imaging of dopamine release in the retina would address several outstanding questions about how dopamine levels are regulated across development and adulthood (Roy and Field, 2019). Here we focused on early postnatal development, when dopamine is released in response to spontaneous retinal waves, which provide patterned activity essential for circuit refinement (Kirkby et al., 2013; Arroyo and Feller, 2016). Dopamine release in the retina is confined to a sparse population of amacrine cells, though recent findings indicate that retinal ganglion cells (RGCs) might transiently release dopamine in a manner that influences vascular development (Liang et al., 2023). Important open questions remain regarding the relative contributions of circadian influences versus melanopsin-expressing ipRGC-driven activity in regulating dopamine release (Zhang et al., 2008; Pérez-Fernández et al., 2019). Developing approaches for real-time dopamine imaging will therefore be essential for defining the sources, timing, and spatial dynamics of dopamine signaling during these critical stages of retinal maturation.

Although beyond the scope of this work, adapting this microscope for in vivo dual-color imaging would be an exciting next step, given the prevalence of two-photon microscopy in in vivo applications. Recent progress integrating nIRCats with fiber photometry (Klinger et al., 2025) suggests a promising direction, though several challenges remain: the signal-to-noise ratio would need to approach that of GRAB_DA_, and nIRCat diffusion, biocompatibility, and clearance in brain tissue would require thorough characterization. Nonetheless, growing interest in near- and far-infrared imaging (e.g., nIR-III window; Feng et al., 2021) underscores the value of these technologies, and the approach described here provides a practical path toward longer-wavelength detection capabilities without constructing an entirely new microscope.

## Synthesis

Reviewing Editor: Matthew Grubb, King's College London Decisions are customarily a result of the Reviewing Editor and the peer reviewers coming together and discussing their recommendations until a consensus is reached. When revisions are invited, a fact-based synthesis statement explaining their decision and outlining what is needed to prepare a revision will be listed below. The following reviewer(s) agreed to reveal their identity: NONE.

Synthesis:

Both Reviewers are very positive about the study, and each make several suggestions for improving the manuscript further. Please explicitly address all of the comments in a revised submission.

Reviewer 1:

In this study, the authors present their development and adaptation of a 2-photon microscope for the live imaging of visible and near IR fluorescence from genetically encoded and carbon nanotube based optical dopamine sensor. The authors describe the adaption of a standard 2 photon microscope, likely used in simultaneous physiology and imaging setups by their group, for the detection of a low affinity dopamine sensor emitting in the near IR spectrum. To show the effectiveness of the imaging adaptation, the authors perform some imaging of visible genetically-encoded dopamine sensor (high DA affinity GRABda), simultaneously with nIRCaT, a low affinity DA sensor in the nIR. Furthermore, the authors provide another proof of principle experiment for this system in the form of imaging of calcium dynamics (Cal520) and dopamine (nIRCat) from the retina. The adaption of the microscope is not described in great detail, but appears to be fairly straightforward. This group clearly has a lot of experience with live imaging of nervous tissue and has the specific "know-how" to construct this microscope adaptation. While this is a useful addition to a sophisticated imaging rig, it is not entirely clear how widely applicable this might be for the majority of labs out there that do not have their own 2ph setup. The study is, nevertheless, well performed and the methodology/analyses are sound. I am not convinced of the usefulness of the nIRCat sensor for dopamine detection in the retina during retinal waves, given its low affinity for dopamine and barely detectable signal above noise. Simultaneous live imaging of two or more fluorescent signals is helpful, but it is hardly novel. Perhaps the better use for nIR imaging would be for sensors that have higher sensitivity than the currently described dopamine sensor, as the authors point out themselves. Overall, this study describes a helpful tool that can be of use to the neuroscience community and is suitable for publication in eNeuro after minor revisions.

Specific comments:

1) Since this is a description of an adaption to an instrument, it seems reasonable to expect that some more specific diagrams and photographs of the microscope conversion should be provided (either as an additional figure, or as a description of the parts list necessary for the conversion).

2) Having additional extended data materials seems unnecessary to me, I would suggest that the authors should include the supplementary data as figures in the main manuscript, especially the extended images from the GRABda and nIRCat imaging experiments.

3) Supplemental figures 2-2, 2-3, 2-4 and 4-1 make a good case for the effectiveness of the imaging adaption in both brain slices and retina. Specifically, the analysis of the time course of rise and decay of the visible and nIR dopamine sensors is nicely done, and provides a good description of the behavior of both sensors in this system, as well as the general behavior of the nIRCaT sensor for dopamine, which seems much noisier and more sluggish.

4) The authors should consider an extended discussion of the potential suitability or adaptability of their modification to nIR sensors with higher affinity.

Reviewer 2:

In the manuscript "Adapting a two-photon scanning microscope for simultaneous single-photon imaging of an infrared dopamine sensor", the authors present a method to couple a fiber to a PMT for one-photon infrared detection within a standard two-photon microscope configuration for simultaneous imaging of green and near-infrared (nIR) dopamine sensors. To validate this method, they performed in vitro imaging in the striatum and retina, combining genetically encoded dopamine sensors (GRABDA) with carbon nanotube-based optical nanosensors (nIRCats). An advantage of this approach is the integration of near-infrared detection in a cost-effective implementation compatible with standard two-photon setups. This optical approach is particularly useful for imaging nIRCats to detect high dopamine concentrations in striatal brain slices, and potentially in other regions like the substantia nigra, using a non-invasive method. However, broader application to regions with low spontaneous release (e.g., retina) may be limited due to the intrinsic low affinity and signal-to-noise ratio (SNR) of nIRCats. There are also a few aspects of data presentation that could be further improved.

Major comments:

1) In my view, the main advantage of the method is to simultaneously image low-affinity nIRCat, which detects high dopamine concentrations, in combination with high-affinity green dopamine sensors under two-photon microscopy. Given that the nIRCat signal is smaller than in previous reports, additional characterization under the present optical configuration would strengthen this point.

a. What are the dynamic range and response linearity of nIRCat? This could be tested using increasing stimulation intensities or dopamine concentrations.

b. Which two-photon excitation wavelengths can be effectively used with nIRCat? This is particularly relevant if the authors emphasize potential compatibility with both green and red sensors.

2) Main figures should include summary data and all essential information.

a. Fig. 2. Overlap is not quantified. Is it possible to provide a quantitative estimate? Tau quantification (Fig. 2-3) should be included in the main figure.

b. Fig. 3. Summary data, including the number of ROIs and normalized dF/F values (currently in Fig. 3-1), should be part of the main figure.

c. Fig. 4. Quantification of nIRCat responses to K+ puffs and retinal waves (currently in Fig. 4-1) is also important for the interpretability of the results.

3) Reporting of sample sizes should be clarified in the figure legends. Please define what experiments and experimental replicates are (e.g., a single stimulus response or the average of multiple stimuli), and report both the number of slices and the number of mice for each figure.

4) Some experiments may lack statistical power (n = 3 brain slices) due to the low SNR and variability of nIRCat signal. For example, Fig. 2D shows higher variability between slices for nIRCat than for GRABDA. It would be helpful to estimate whether this variability reflects differences within experiments, slices of the same preparation, or across animals.

5) Statistical analyses should be performed for the following comparisons: Fig. 3B, D; Fig. 2-3C, E; Fig. 3-1; and Fig. 4-1.

Minor comments:

1) Lines 81-82: Note that neonatal mice (P4-P8) were used for retina preparations. Please also indicate the minimum age for adult animals (e.g., >3 months).

2) Lines 116-130. If nIRCat synthesis and labeling methodology were adapted from a previous study, please cite accordingly.

3) Line 183. I may have misunderstood Figure 1A. Could the authors clarify whether one-photon excitation was used? A fluorescence profile of nIRCat across a two-photon range (e.g., 850-1100 nm) would also be informative. See also major comment 1.

4) Lines 206-207. Axes of Fig. 1C lack units or labels. If the x-axis represents axial resolution, the estimated FWHM seems smaller than the reported 100 µm and 50 µm values for each corresponding fiber. Please clarify.

5) Lines 228-230 (Fig. 2 and Fig. 2-2): Is there an explanation for the distinct expression gradients observed between GRABDA and nIRCat that may not be fully explained by cellular density?

6) Lines 235-237. Would a comparison of mean amplitudes between GRABDA and nIRCat be more informative? Or is the goal of Fig. 2D to show a positive correlation between the signals of both sensors?

7) Line 246. Related to Figure 2-4B. Same comment as above.

8) Lines 273-275: Please clarify whether the same electrically evoked stimulation paradigm was used for the experiments shown in Fig. 3.

9) Lines 370-373. Without characterization of nIRCat responses at the longer excitation wavelengths required for dual imaging of green and red sensors under two-photon conditions, the authors may consider qualifying this statement (see major comment 1).

10) Lines 404-406. In its current form, the applicability of this technology to in vivo experiments appears limited. A brief discussion of technical challenges that must be addressed would be useful.

11) Missing panel letters in Figure 2-1.

## Author Response

Dear Dr. Grubb We are pleased to submit the revision of our manuscript, "Adapting a two-photon scanning microscope for simultaneous single-photon imaging of an infrared dopamine sensor," for consideration as a Research Article in eNeuro.

Thank you for handling the manuscript, passing on the reviews and providing a clear synthesis statement of the reviewer comments. We were happy to read that reviewers were positive about the study. Below, we provide a point-by-point response to each reviewer's comment, detailing the revisions made to the manuscript. The reviewer comments are reproduced in black, our responses are in blue, and we reference all the revised text by line number. In the revised text, we have highlighted the changes. Note, we have also reformatted many of the figures to accommodate the supplemental figures, as requested by the reviewers.

To address the code accessibility issue you raised, there is now a section in Materials and Methods (Lines 194-201 in revised text):

Code accessibility Python-based image processing and analysis applications are available at https://github.com/NicholasOuassil/NanoImgProand https://github.com/FellerLabCodeShare/nIR-catecholamine-sensorhttps://github.com/FellerLabCodeShare/nIR-catecholamine-sensor. The code is publicly accessible and does not require special permissions; no accession number is applicable. Analyses were performed using Python (version 3.12.12) on macOS- based systems and in cloud-based environments using Google Colaboratory. The code was executed on standard workstation hardware, and required packages and installation instructions are provided in the repository.

RESPONSE TO REVIEWER COMMENTS: Reviewer 1:

In this study, the authors present their development and adaptation of a 2-photon microscope for the live imaging of visible and near IR fluorescence from genetically encoded and carbon nanotube based optical dopamine sensor. The authors describe the adaption of a standard 2 photon microscope, likely used in simultaneous physiology and imaging setups by their group, for the detection of a low affinity dopamine sensor emitting in the near IR spectrum. To show the effectiveness of the imaging adaptation, the authors perform some imaging of visible genetically-encoded dopamine sensor (high DA affinity GRABda), simultaneously with nIRCaT, a low affinity DA sensor in the nIR. Furthermore, the authors provide another proof of principle experiment for this system in the form of imaging of calcium dynamics (Cal520) and dopamine (nIRCat) from the retina. The adaption of the microscope is not described in great detail, but appears to be fairly straightforward. This group clearly has a lot of experience with live imaging of nervous tissue and has the specific "know-how" to construct this microscope adaptation. While this is a useful addition to a sophisticated imaging rig, it is not entirely clear how widely applicable this might be for the majority of labs out there that do not have their own 2ph setup. The study is, nevertheless, well performed and the methodology/analyses are sound. I am not convinced of the usefulness of the nIRCat sensor for dopamine detection in the retina during retinal waves, given its low affinity for dopamine and barely detectable signal above noise. Simultaneous live imaging of two or more fluorescent signals is helpful, but it is hardly novel.

Perhaps the better use for nIR imaging would be for sensors that have higher sensitivity than the currently described dopamine sensor, as the authors point out themselves. Overall, this study describes a helpful tool that can be of use to the neuroscience community and is suitable for publication in eNeuro after minor revisions.

We thank the reviewer for the positive comments on the soundness of the study. They have made several suggestions that might make their results more useful for the general public that we have incorporated.

Specific comments:

1) Since this is a description of an adaption to an instrument, it seems reasonable to expect that some more specific diagrams and photographs of the microscope conversion should be provided (either as an additional figure, or as a description of the parts list necessary for the conversion).

We have modified Figure 1 to accommodate the comments of both reviewers. To address this comment specifically we have added a photo in the new Figure 1A. We also have a complete parts list at the github site associated with the study (Lines 145-147).

2) Having additional extended data materials seems unnecessary to me, I would suggest that the authors should include the supplementary data as figures in the main manuscript, especially the extended images from the GRABda and nIRCat imaging experiments.

Please see our response below for Comment 3.

3) Supplemental figures 2-2, 2-3, 2-4 and 4-1 make a good case for the effectiveness of the imaging adaption in both brain slices and retina. Specifically, the analysis of the time course of rise and decay of the visible and nIR dopamine sensors is nicely done, and provides a good description of the behavior of both sensors in this system, as well as the general behavior of the nIRCaT sensor for dopamine, which seems much noisier and more sluggish.

We appreciate the reviewer's comment that this analysis is valuable. We have now accommodated both Comments 2 and 3 in the following changes: Specifically, ● Figure 2-1 and 2-4: incorporated to revised Figure 2 ● Figure 2-3: now revised Figure 3 ● Figure 3-1: incorporated to revised Figure 4 ● Figure 4-1: incorporated to revised Figure 5 We have also included an additional description in the revised text (Lines 247-268).

4) The authors should consider an extended discussion of the potential suitability or adaptability of their modification to nIR sensors with higher affinity.

To our knowledge, the nIRCats used in this study have the highest affinity for dopamine sensors available in the near-infrared range (> 1000 nm). However, we have revised the text (Lines 353-357) to note that other infrared sensors could potentially be integrated with our system.

Reviewer 2:

In the manuscript "Adapting a two-photon scanning microscope for simultaneous single-photon imaging of an infrared dopamine sensor", the authors present a method to couple a fiber to a PMT for one - photon infrared detection within a standard two-photon microscope configuration for simultaneous imaging of green and near-infrared (nIR) dopamine sensors. To validate this method, they performed in vitro imaging in the striatum and retina, combining genetically encoded dopamine sensors (GRABDA) with carbon nanotube-based optical nanosensors (nIRCats). An advantage of this approach is the integration of near-infrared detection in a cost-effective implementation compatible with standard two- photon setups. This optical approach is particularly useful for imaging nIRCats to detect high dopamine concentrations in striatal brain slices, and potentially in other regions like the substantia nigra, using a non-invasive method. However, broader application to regions with low spontaneous release (e.g., retina) may be limited due to the intrinsic low affinity and signal-to-noise ratio (SNR) of nIRCats. There are also a few aspects of data presentation that could be further improved.

We thank the reviewer for the positive comments on the usefulness of the approach. They have made several suggestions that might make our results more broadly useful and improve the presentation of the data.

Major comments:

1) In my view, the main advantage of the method is to simultaneously image low-affinity nIRCat, which detects high dopamine concentrations, in combination with high-affinity green dopamine sensors under two-photon microscopy. Given that the nIRCat signal is smaller than in previous reports, additional characterization under the present optical configuration would strengthen this point. a. What are the dynamic range and response linearity of nIRCat? This could be tested using increasing stimulation intensities or dopamine concentrations.

The dynamic range and linearity of these sensors have been previously reported. We have now included these references in Lines 250-251.

For the benefit of the reviewer, we conducted these experiments for one brain slice. Since we only conducted this for one brain and it was consistent with previous studies, we did not include these data in the revision. Hill equation is defined as Δ*F*/*F*0=(Δ*F*/*F*0)*m**a**x*⋅([*D**o**p**a**m**i**n**e*]*K**d*+[*D**o**p**a**m**i**n**e*])*n*, where *K**d* is the dissociation constant and *n* is the Hill slope factor. We obtained (Δ*F*/*F*0)*m**a**x*= 0.028, *K**d* = 5.4 μM, and *n* = 1.4 as fitting parameters. From here, the functional dynamic range (10-90% response) was calculated using the Hill inversion formula: [*D**o**p**a**m**i**n**e*]=*K**d*⋅(Δ*F*/*F*0(Δ*F*/*F*0)*m**a**x*− Δ*F*/*F*0)1*n*= 5.4⋅(Δ*F*/*F*00.028 − Δ*F*/*F*0)11.4 and determined to be 1 μM to 27 μM. The response was approximately linear between 1-10 μM dopamine (R² ≈ 0.83), as determined by linear regression of ΔF/F0 versus concentration (Figure R1B).

Figure R1. A. ∆F/F0 of nIRCat as a function of dopamine concentration. The data were fitted with the Hill equation (dashed line; R2 = 0.90), and the dissociation constant Kd was 5.4 µM. B. Linear regression fit (dash line; R2 = 0.83) in the low concentration range. b. Which two-photon excitation wavelengths can be effectively used with nIRCat? This is particularly relevant if the authors emphasize potential compatibility with both green and red sensors.

In our studies, we are using 1-photon excitation of nIRCat using a laser tuned to the infrared (in this study, 920-950 nm). nIRCat can also be excited with wavelengths ranging from 600 -1000 nm, due to heterogeneity in the chirality of the sensors during synthesis. (Please see response to Minor Comment #3 and Figure R2 below). We have not made any changes to the text based on this comment.

2) Main figures should include summary data and all essential information. a. Fig. 2. Overlap is not quantified. Is it possible to provide a quantitative estimate? Tau quantification (Fig. 2-3) should be included in the main figure.

We did not quantify the overlap because it does not have biological implications (GRABDA expression is in axon terminals in the striatum, TH labels dopamine axon terminals, and the bath-applied nIRCat, resides in the extracellular space). In addition, our low magnification imaging does not have the resolution to describe overlap at the cellular level.

Tau quantification is now provided in new Figure 3. We have presented a description of this new finding in the revised text (Methods: Lines 172-176; Results: Lines 259-268). b. Fig. 3. Summary data, including the number of ROIs and normalized dF/F values (currently in Fig. 3 - 1), should be part of the main figure.

The previous Figure 3-1 is now incorporated into the revised Figure 4. c. Fig. 4. Quantification of nIRCat responses to K+ puffs and retinal waves (currently in Fig. 4-1) is also important for the interpretability of the results.

Quantification of the nIRCat response to K+ puffs and retinal waves (previously in Figure 4-1) is now incorporated into the revised Figure 5.

3) Reporting of sample sizes should be clarified in the figure legends. Please define what experiments and experimental replicates are (e.g., a single stimulus response or the average of multiple stimuli), and report both the number of slices and the number of mice for each figure.

We have provided this information in the revised figure captions.

4) Some experiments may lack statistical power (n = 3 brain slices) due to the low SNR and variability of nIRCat signal. For example, Fig. 2D shows higher variability between slices for nIRCat than for GRABDA. It would be helpful to estimate whether this variability reflects differences within experiments, slices of the same preparation, or across animals.

The dataset provided in Figure 2G (previously Figure 2D) and 2H directly compared the simultaneous fluorescence change for nIRCat and GRABDA evoked by stimulation of dopaminergic axon terminals, and there is variance across the preparations due to differences in the effectiveness of the stimulation, the expression of GRABDA, and the concentration of the nIRCat. We observed that the nIRCat ΔF/F0 monotonically increases with GRABDA ΔF/F0, suggesting that the nIRCat ΔF/F0 reflects physiological changes in dopamine concentrations between brain slices or regions where the electrode was placed. The higher variability of the nIRCat ΔF/F0 is likely due to the lower signal-to-noise than the GRABDA.

To clarify this point, we revised the text (Lines 253-255).

5) Statistical analyses should be performed for the following comparisons: Fig. 3B, D; Fig. 2 -3C, E; Fig.

3-1; and Fig. 4-1.

We have performed the statistical analysis, and the results are included in the revised figure captions. Note, for Figure 2-3 C and E (now incorporated into Figure 3), in all examples, nIRCat kinetics were slower than the matched pairs for GRAB-DA. However, given the large variance in the time courses we observed, our results were underpowered to make a meaningful statistical comparison.

Minor comments:

1) Lines 81-82: Note that neonatal mice (P4-P8) were used for retina preparations. Please also indicate the minimum age for adult animals (e.g., >3 months).

We have added the ages in the revised Methods.

2) Lines 116-130. If nIRCat synthesis and labeling methodology were adapted from a previous study, please cite accordingly.

We made following changes in the methods (Lines 119-120): nIRCat synthesis: nIRCats were prepared following previously developed protocolhttps://www.zotero.org/google-docs/?oa5yYD. Briefly, HiPCo SWCNT slurry (NanoIntegris) and ssDNA (Integrated DNA Technologies) were mixed with a final NaCl concentration of 10 mM.

3) Line 183. I may have misunderstood Figure 1A. Could the authors clarify whether one -photon excitation was used? A fluorescence profile of nIRCat across a two-photon range (e.g., 850-1100 nm) would also be informative. See also major comment 1.

Our strategy is to use single-photon excitation for nIRCats and two-photon excitation for the GFP-based sensors. We realize now that our initial figure did not include absorption > 800 nm, which has led to this confusion. There are multiple studies showing that single-walled-carbon nanotubes have photoluminescence in these wavelengths - one example is shown below (Figure R2). We are not sure if it is helpful (or appropriate) to include such a panel in our figure. Therefore, we have removed the original photoluminescence map and described the broad excitation spectra of these SWCNT sensors in the text (lines: 204-205).

Figure R2. Carbon nanotube chiralities (denoted as (n, m)) with their resonant excitation and emission wavelength (note that they can also be excited off-resonance). The wavelength range used in this study is highlighted in red. Adapted from (https://pubs.rsc.org/en/content/articlelanding/2025/nh/d5nh00231a) 4) Lines 206-207. Axes of Fig. 1C lack units or labels. If the x-axis represents axial resolution, the estimated FWHM seems smaller than the reported 100 µm and 50 µm values for each corresponding fiber. Please clarify.

We have added the units (µm) to Figure 1C. We display only half of the FWHM in this figure, which is why it looks smaller.

5) Lines 228-230 (Fig. 2 and Fig. 2-2): Is there an explanation for the distinct expression gradients observed between GRABDA and nIRCat that may not be fully explained by cellular density? Sometimes slices were not completely flat and therefore for the green channel, part of the slice would be out of focus. nIRCat loading was not always uniform. We address this in Lines 239-240.

6) Lines 235-237. Would a comparison of mean amplitudes between GRABDA and nIRCat be more informative? Or is the goal of Fig. 2D to show a positive correlation between the signals of both sensors? The point was to show a positive correlation between both sensors. We have revised the text to address this point (Lines 247-255). Please also see our answer to Major Comment 4.

7) Line 246. Related to Figure 2-4B. Same comment as above.

Please see our answer to the comment above (Comment 6 and Major Comment 4).

8) Lines 273-275: Please clarify whether the same electrically evoked stimulation paradigm was used for the experiments shown in Fig. 3.

It was the same electrically evoked stimulation. It has been clarified in the Revised Figure 4 captions.

9) Lines 370-373. Without characterization of nIRCat responses at the longer excitation wavelengths required for dual imaging of green and red sensors under two-photon conditions, the authors may consider qualifying this statement (see major comment 1).

We have removed what was admittedly an overly complicated imaging scenario. Rather, we have emphasized the use of red sensors that could be utilized in this scope design (Lines 353-357).

10) Lines 404-406. In its current form, the applicability of this technology to in vivo experiments appears limited. A brief discussion of technical challenges that must be addressed would be useful.

We have extended this discussion in the revised text (Lines 390-393) 11) Missing panel letters in Figure 2-1.
